# Kinematic characteristics of second‐order motor planning and performance in 6‐ and 10‐year‐old children and adults: Effects of age and task constraints

**DOI:** 10.1002/dev.21911

**Published:** 2019-09-09

**Authors:** Erik Domellöf, Anna Bäckström, Anna‐Maria Johansson, Louise Rönnqvist, Claes von Hofsten, Kerstin Rosander

**Affiliations:** ^1^ Department of Psychology Umeå University Umeå Sweden; ^2^ Department of Psychology Uppsala University Uppsala Sweden

**Keywords:** action prediction, children, end state comfort, kinematics, motor planning

## Abstract

This study explored age‐related differences in motor planning as expressed in arm‐hand kinematics during a sequential peg moving task with varying demands on goal insertion complexity (second‐order planning). The peg was a vertical cylinder with either a circular or semicircular base. The task was to transport the peg between two positions and rotate it various amounts horizontally before fitting into its final position. The amount of rotation required was either 0°, 90°, 180°, or −90°. The reaching for the peg, the displacement of it, and the way the rotation was accomplished was analyzed. Assessments of end state comfort, goal interpretation errors, and type of grip used were also included. Participants were two groups of typically developing children, one younger (*M*_age_ = 6.7 years) and one older (*M*_age_ = 10.3 years), and one adult group (*M*_age_ = 34.9 years). The children, particularly 6‐year‐olds, displayed less efficient prehensile movement organization than adults. Related to less efficient motor planning, 6‐year‐olds, mainly, had shorter reach‐to‐grasp onset latencies, higher velocities, and shorter time to peak velocities, and longer grasp durations than adults. Importantly, the adults rotated the peg during transport. In contrast, the children made corrective rotations after the hand had arrived at the goal.

## 1 INTRODUCTION

In everyday life, we perform a variety of prehensile activities that extend beyond reaching out and grasping an object, for example, taking a memory stick to plug it into a computer port. Thus, most of the time, we plan these actions to accomplish an onward final goal (so called second‐order motor planning). Once an object has been grasped, it is also often necessary to adjust or rotate the object during the transport phase to optimize the object fit in relation to the final location, and to the configuration of the object. While extensively studied at different ages in relation to the end state comfort effect (ESC; Rosenbaum, Chapman, Weigelt, Weiss, & van der Wel, 2012), less is known about the spatio‐temporal parameters underlying this important ability. In this study, the kinematics of “reach‐to‐grasp” and “transport‐to‐fit” (accurate online fitting of an object into a goal slot) performance are evaluated, adding a kinematic characterization of second‐order motor planning and performance in 6‐year‐olds (an age when children typically show limited planning ability in ESC tasks, cf. Scharoun Benson, Roy, & Bryden, 2018) and 10‐year‐olds (typically showing practically adult‐like planning ability in ESC tasks, cf. Scharoun Benson et al., 2018) compared with adults.

Goal‐directed actions, such as reach‐to‐grasp movements, are typically regulated by both feed‐forward and feedback control mechanisms, the former responsible for proactive movements based on previous sensorimotor experience and assumptions of the world, the latter for movement corrections based on sensory responses (Flanagan, Bowman, & Johansson, 2006; Glover, 2004). Action prediction is essential for efficient motor control as movements only depending on reactive feedback mechanisms would lead to slow and clumsy motor performance (Kawato & Gomi, 1992; von Hofsten, 2014). Motor planning refers to the ability to predict an action goal and to organize the motor behaviors required to attain it. Such anticipatory processing may also involve several phases. For instance, first‐order motor planning involves adjusting motor behavior toward an imminent goal, for example, grasping an object. Second‐order motor planning involves the adjustment of motor behavior not only toward the imminent goal but also toward the goal of the subsequent motor task, for example, placing the apprehended object in a container (Rosenbaum et al., 2012).

Future‐oriented manual actions emerge early in development. At the beginning of successful reaching at about 4 months of age, infants anticipate movement goals in terms of hand orientation (von Hofsten & Fazel‐Zandy, 1984) and hand opening (von Hofsten & Rönnqvist, 1988). Moreover, infants also show emerging second‐order motor planning. For instance, one study (Claxton, Keen, & McCarty, 2003) reported that 10‐month‐old infants displayed a slower approach of an object to be fitted into a tube than for throwing it into a tub. Differences in reaching kinematics depending on degree of precision required for a goal‐directed block task have also been reported in toddlers at 18–21 months (Chen, Keen, Rosander, & von Hofsten, 2010). Toddlers had longer deceleration as the hand approached the block for pickup in a precise task (building towers) compared with an imprecise task (placing it in an open container). Furthermore, by 22 months of age, toddlers have been observed to adjust the orientation of a block to be fitted into an aperture by predictively adjusting its orientation as the hand reaches the aperture (Örnkloo & von Hofsten, 2007). Kinematic studies in young children have additionally reported a developmental progress in coordinating translations and rotations of handled objects to be fitted into apertures. The results suggest an improved spatial planning ability over the toddler years (Jung, Kahrs, & Lockman, 2015, 2018).

Thus, motor planning is a fundamental ability and any disruption to the developing action prediction during childhood would cause problems in daily life activities. Still, knowledge is limited regarding the detailed characterization of action prediction in the middle childhood years (about 4–12 years). Several previous studies have reported largely consistent findings of age‐related advances in prehensile movement performance during middle childhood, also in reference to adult performance (Kuhtz‐Buschbeck, Stolze, Jöhnk, Boczek‐Funcke, & Illert, 1998; Olivier, Hay, Bard, & Fleury, 2007; Schneiberg, Sveistrup, McFadyen, McKinley, & Levin, 2002; Simon‐Martinez et al., 2018), characterized by increased velocity, straighter reaching trajectories, increased smoothness, and less variability. Thus, young children at 5–7 years of age display relatively immature manual visuomotor coordination and control, eventually reaching developmental stability in terms of spatiotemporal parameters at 11–12 years of age (although not yet at an adult level).

Improvements in goal‐directed upper‐limb movement organization have been suggested related to the development of motor planning (Simon‐Martinez et al., 2018), also supported by observations of a parallel developmental trajectory for motor planning abilities between 3 and 12 years of age according to the end state comfort effect (ESC; Jongbloed‐Pereboom, Nijhuis‐van der Sanden, Saraber‐Schiphorst, Crajé, & Steenbergen, 2013; Rosenbaum et al., 2012; Scharoun Benson et al., 2018; Stöckel, Hughes, & Schack, 2012; van Swieten et al., 2010; Wilmut & Byrne, 2014; Wunsch, Pfister, Henning, Aschersleben, & Weigelt, 2016). A few studies have investigated planning aspects within the framework of goal‐directed reach‐to‐grasp kinematics in children, although with some inconsistency. For instance, with regard to variation in object sizes (i.e., requiring more or less precision), Kuhtz‐Buschbeck et al. (1998) reported no influence of object size on movement duration and velocity in children at 4–12 years old, and that only the oldest children showed a precise grip formation depending on object size. Another study noted that object size did influence movement kinematics in 5‐year‐old children, but children did not show the expected lowered peak velocity amplitude for smaller compared with larger objects (Zoia et al., 2006). Regarding second‐order planning ability, Wilmut, Byrne, and Barnett (2013) found differences in initial reach‐to‐grasp kinematics depending on type of onward action in children at 4–11 years. The youngest children (4–5 years) displayed increased movement duration for placing an object with precision compared with throwing it. Depending on complexity of onward action, older children showed discrimination in terms of decelerating time, although not at an adult level.

While these findings are promising, there is a need for additional detailed analysis of goal‐directed manual movements to better understand the planning of motor movements over the middle childhood years, in particular with regard to movements involving several sub‐goals. To solve such a task efficiently, the child needs to plan a sequence of movements in advance, also considering adjustments that may be necessary to accomplish the global goal. Given the vast amount of goal‐directed sequential manual actions that are required in everyday life, surprisingly little is known about the spatio‐temporal representation of the planning of such actions in children in the preschool and school years. Apart from the theoretical interest, such improved understanding of typical action development is alsoimperative to guide early diagnosis of motor performance deficits and potential intervention practices. This study aimed to investigate differences in sequential manual motor planning between two age groups of typically developing children, one younger (mean age 6 years) and one older (mean age 10 years), and an adult reference group in terms of movement kinematics during performance of a peg moving task with varying task demands. The peg was a vertical cylinder that was either circular or semicircular. The task was to move the peg between two positions and, if required, rotate it various amounts horizontally before fitting into its final position. The most optimal way to do this was to grasp the peg in a way that anticipates its future rotation, rotate the peg inside the hand, and coordinate the translation and rotation to be completed at the goal slot. Not rotating the peg inside the hand when required would necessitate an extensive whole arm/upper‐body rotation to be able to fit the peg into the goal slot, that is, an uncomfortable end state posture (cf. Rosenbaum et al., 2012). The reaching for the peg, the displacement of it, and how the rotation was accomplished were analyzed.

The global movement required in this task was divided into four phases: a latency phase (from goal becoming visible to start ofmovement), a reach‐to‐grasp phase (from the start of movement to arrival at the peg), a grasp phase (from arrival at the peg to lifting of it, i.e., a period of grip formation), and a transport‐to‐fit phase (from lifting the peg to fitting it into the goal slot). Compliant with the planning‐control model for goal‐directed reaching movements (Glover, 2004), the first phase (latency) represents the premovement planning stage that depends exclusively on planning processes, the two subsequent phases (reach‐to‐grasp and grasp) can be assumed relying mainly on initial planning processes, and the last phase (transport‐to‐fit) as more influenced by control processes, with corresponding impact on kinematic movement parameters during the different parts of the movement. On the basis of previous research, we generally expected to find reliable differences in kinematic outcomes depending on age group. More specifically with regard to motor planning, the following main research questions were pursued:

1. Are there age‐related differences in kinematic outcome of a peg fitting task during the planning of initial movement sequences (latency, reach‐to‐grasp and grasp)?
2. Are there age‐related kinematic differences depending on task constraints during the transport‐to‐fit phase?
3. Was the main rotation of the peg accomplished before or after the arrival at the goal?
4. Are there associations between movement organization during the reach‐to‐grasp and transport‐to‐fit phases that demonstrate planning, and how do they vary depending on age?

## 2 METHOD

### 2.1 Participants

Within the framework of an ongoing study of motor planning ability in children, eight children at 6 years (four girls; mean age = 6.7 years, range: 6.2–7.5), eight children at 10 years (three girls; mean age = 10.3 years, range: 9.9–10.4), and eight healthy adults (four females; mean age = 34.9 years, range: 26.5–42.4) were recruited as participants. The children were recruited through advertisement in a school located close to the local university (*n* = 8) and by convenience sampling (*n* = 8). The adults were recruited at the university. The handedness of the younger children was determined by parent ratings based on an age‐modified version of the Edinburgh handedness questionnaire (Oldfield, 1971). The handedness of the older children and the adults was determined based on writing hand. One of the younger children and one adult were left‐handed. All participants gave their assent to participate in the study. The adults and the parents of the children signed an informed consent form prior to participation. The study was approved by the Umeå Regional Ethical Board (registration nr 2016/365‐31) and conducted in accordance with the Declaration of Helsinki.

### 2.2 Measures and procedure

**Figure 1:**
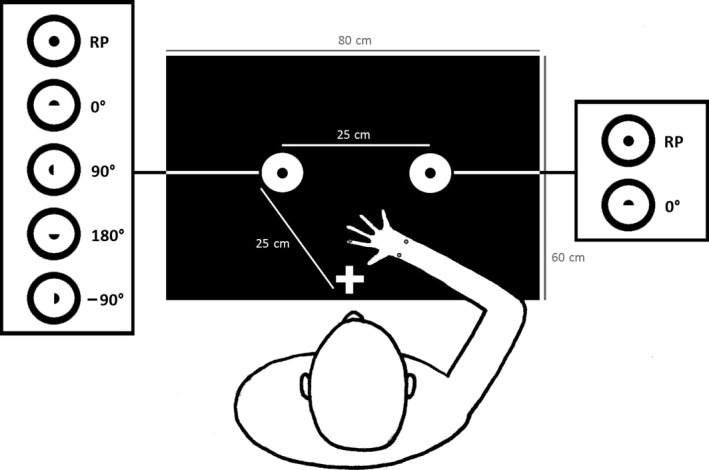
Illustration of the experimental set‐up in a bird's eye view, including marker placement and the different start and goal conditions. The peg is positioned in the start holder to the right (for a right‐handed participant), about to be grasped, transported and fitted into the goal‐holder (to the left). Abbreviation: RP, round peg

Each participant was seated in front of a testing table (length 60 cm, width 80 cm, height 72 cm). The height of the chair and distance from the table was individually adjusted to ensure comfortable task performance. The experimental set‐up is illustrated in Figure 1. The participants performed a sequential goal‐directed task where they were asked to transport a round, cylindrical peg (diameter: 2.5 cm) or a semi‐circular peg (diameter line: 2.5 cm) from a start‐holder to a goal‐holder. For the round peg (RP), the start‐ and goal‐holder were equal (baseline condition). In the semi‐circular condition, the goal‐holder was presented in four different orientations (0°, 90°, 180°, −90°) relative to the frontoparallel axis, thus, introducing different constraints on action planning. The distance between the start‐ and goal‐holder centers was 25 cm. The goal‐holder was initially occluded by a black screen and the goal was only revealed when the experimenter removed the screen at the start of measurement. Participants were asked to start when they saw the goal. After a practice round, allowing familiarization of the material and of moving the peg in the different orientations, all participants performed two blocks of each of the five conditions, with both left and right hand, in a randomized order (20 trials in total). The preset trial measurement time was 10 s for the younger children and 6 s for the older children and adults. Unsuccessful trials during either block, for example, due to dropping the peg or taking the peg with the wrong hand, were repeated at the end of the block. In this study, results from the preferred hand were analyzed.

Movements were recorded by a 6‐camera optoelectronic system with a sampling frequency of 120 Hz (Oqus, Qualisys Inc.). Two cameras were placed in front of the testing table at a distance of about 1.4 m, and four were attached to a rail in the roof about 1.7 m above the calibrated space. Spherical passive markers were fixed to the index finger (7 mm) and to the left (radial styloid) and right (ulna styloid) side of the wrist (12 mm) on both hands of the participants. The peg was equipped with two flat circular markers (5 mm) on each side of the top (distance: 2 cm). The peg was further equipped with a green tape around the center of the sides, indicating where the participants should grasp the peg (i.e., the participants were not allowed to grasp the peg at the top). One flat round marker (5 mm) was also imbedded in the target hole of the goal‐holder, functioning as an indicator for the time point when the goal was visible to the participant. A web camera, situated about 1.4 m in front of the child, collected additional information.

### 2.3 Kinematic analysis

The Qualisys system software was preset to gap‐fill small occlusions of the markers (maximum 10 frames). These automatic gap‐fills were inspected and removed if deemed incorrect. If possible to perform reliably, larger gap‐fills (maximum 20 frames) were manually filled and accepted after visual inspection in the three different planes of space and the velocity profile. A total of 78 trials for the 6‐year‐olds, 78 for the 10‐year‐olds, and 80 for the adults were included in the analyses. All data were smoothed using a second‐order 12 Hz Butterworth filter.

**Figure 2:**
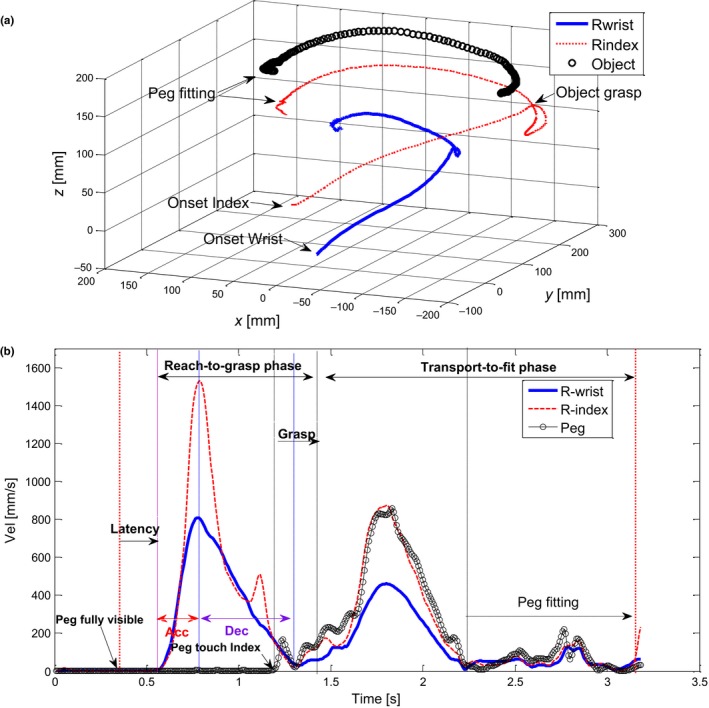
Illustration of (a) the 3D motion paths of the wrist, index finger and the object/peg during a reach‐to‐grasp‐to‐fit trial made by a 10‐year‐old child in the 180° task condition, and (b) the corresponding velocity profiles including descriptions of movement phases and parameters of interest. Note: rotation parameter Rota I is linked to the peg transport phase (from grasp to peg fitting), and Rota II to the peg fitting phase

The latency was defined as the difference between the frame where the goal marker appeared and movement onset as determined by the frame where the tangential velocity of the primary wrist marker attained or exceeded 20 mm/s. One kinematic parameter of interest was the spatio‐temporal segmentation of the movement path, hence, the number of movement units (MU) were extracted. A MU was defined as an accumulated increase and decrease in velocity of at least 20 mm/s with an acceleration or deceleration exceeding 5 mm/s^2^ (von Hofsten, 1991). In order to avoid overlooking any initial MU, the criterion used for defining the onset of the reach‐to‐grasp phase was five frames before the frame where the tangential velocity of the wrist marker attained or exceeded 20 mm/s. The criterion used for defining the end of the reach‐to‐grasp phase was five frames after the tangential velocity of the wrist marker had reached a low point at the end of the approach (i.e., including any final MU), with a simultaneous change in tangential velocity of the object markers (i.e., indicating touch of peg). The criterion used for defining the onset of the transport‐to‐fit phase was five frames before the peg marker moved 1 mm upwards. End of transport‐to‐fit was defined as the frame when the tangential velocity of the wrist marker attained or exceeded 60 mm/s in the process of returning the hand to the starting point (i.e., after the peg had been fitted into the goal slot). The grasp phase was defined as the difference between the end of reach‐to‐grasp and beginning of transport‐to‐fit excluding the corrections of five frames. Figure 2 provides an illustration of the 3D motion paths (including wrist, index finger and the object/peg) and the corresponding velocity profiles (including parameters of interest for statistical analyses) during a full trial (all phases).

Kinematic data for the reach‐to‐grasp and transport‐to‐fit phases were extracted by customized MATLAB (The Mathworks Inc.) scripts. The following parameters were calculated: movement duration, average velocity, amplitude of peak velocity, movement segmentation in terms of number of MUs, and peak velocity placement percentage (PPV; defined as the percentage of movement time where the peak velocity occurs).

### 2.4 Peg rotation

To accomplish the fitting of the peg, its orientation must be rotated accordingly to the orientation of the goal slit simultaneously with the translation. Alternatively, if the orientation is not a part of the movement plan, the rotation of the peg could be adjusted at the goal. In this study, the amount of rotation and its duration were calculated from the kinematic data. The rotation of the peg during transport‐to‐fit consisted of a main rotation during the transport (Rota I) combined with corrective rotations at the goal (Rota II). Using customized MATLAB scripts, the calculations were focused on these two movements. As start and goal were defined along a horizontal line in the frontal plane, the rotation of the peg was calculated for the horizontal component of the movement. The difference between the coordinates of the two markers on the peg constituted a horizontal line on the top of the peg that defined the angle relative to the frontoparallel axis. At the end of the transport, the angle of the horizontal line of the peg relative the frontoparallel axis for the time of arrival at the goal (Rota I) was calculated. In addition, the time of the corrective rotation after arrival at the goal to fitting of the peg was calculated (Rota II).

### 2.5 Statistical analysis

For the kinematic outcome measures, separate mixed design 3 × 5 (age groups: 6‐year, 10‐year, adults × task conditions: RP, 0°, 90°, 180°, −90°) ANOVAs were used to analyze the kinematic outcome values of each parameter of interest for the latency‐, reach‐to‐grasp‐, transport‐to‐fit, and rotation phases, by STATISTICA software. All kinematic data were initially tested and verified for normal distribution and homogeneity of variance. Post hoc follow‐up comparisons were systematically performed where a main effect or a significant interaction was observed using the Scheffé post hoc test. Analyses of relations between parameters were performed separately within each age group using Pearson's product‐moment and partial correlations (with Bonferroni correction applied, considering individual tests at *p* < .005 to be significant). Due to multiple comparisons among variables (groups, tasks, and movement phases), the threshold for significance testing was set to *p* ≤ 0.005 for all main and interaction effects, following the recommendation by Benjamin et al. (2018). For post hoc comparisons, an alpha level of 0.01 was used.

### 2.6 Video coding

In order to get a general impression of planning behavior to support the kinematic analyses, video recordings of each trial of the participants were coded for ESC, goal interpretation error, and type of grip according to the following rating procedure: ESC (yes, no) was determined for the 90°, 180°, and −90° conditions as judged by whether the participant showed ESC (rotating peg in hand during transport‐to‐fit, not displaying augmented body movements when fitting the peg into the goal‐holder) or not (rotating arm and hand at the end of transport‐to‐fit and displaying augmented body movements in order to fit the peg into the goal‐holder). Goal interpretation error (yes, no) was deemed present if the participant did not consider/misinterpreted the secondary goal (i.e., whether the goal was rotated 0°, 90°, 180°, −90°) and ended up with the peg in an erroneous end rotation (e.g., 0° instead of 180°). Type of grip (efficient, inefficient) was coded as digit grip (three fingers or more, typically positioned at the edges of the semi‐circular peg allowing comfortable rotation), pincer grip (two fingers, typically thumb and index positioned at the back and front of the semi‐circular peg) or cylindrical/power grip, with the foremost type categorized as efficient and the two latter types combined to form an inefficient category. Inter‐judge reliability (Cohen's kappa), obtained from two judges (ED, AB) independently scoring three random trials of all participants (72 trials in total; 30% of all trials), was 1.0 for ESC, 0.74 for goal interpretation error, and 0.85 for type of grip.

## 3 RESULTS

### 3.1 Kinematic outcomes

Table 1 presents the kinematic outcome parameters for the different phases (latency, reach‐to‐grasp, grasp, transport‐to‐fit) with corresponding age group means (adult, 10‐ and 6‐years) and main effects of group and task (*F*‐, *p*‐, and partial eta squared [*η* ^2^ *p*] values) for each variable.

**Table 1:** Means and standard errors for kinematic outcomes as a function of age group together with main effects of age and task

Kinematic parameters	Age group				
	Adult	10‐year	6‐year	Main effect of age	Main effect of task
Latency phase					
Wrist onset latency (ms)	**296 ± 25.7** ^a^	**128 ± 30.3** ^b^	187 ± 25.2	*F* = 9.3, *p* < .001, *η* ^2^ *p* = 0.08	*F* = 1.1, *p* = .39, n.s.
Reach‐to‐grasp phase					
Reach duration (ms)	846 ± 30.4	733 ± 34.1	**905 ± 30.4** ^c^	*F* = 6.5, *p* < .005, *η* ^2^ *p* = 0.05	*F = *1.5,* p* = .18, n.s.
Wrist MUs (*n*)	1.3 ± 0.1	1.7 ± 0.1	**2.4 ± 0.1** ^c^	*F* = 15.7, *p* < .001, *η* ^2^ *p* = 0.12	*F* = 1.3, *p* = .25, n.s.
Index MUs (*n*)	3.1 ± 0.2	3.2 ± 0.2	3.4 ± 0.2	*F* = 0.7, *p* = .51, n.s.	*F* = 0.8, *p* = .48, n.s.
Wrist peak velocity (mm/s)	**654 ± 20.9** ^a^	942 ± 20.7	852 ± 20.9	*F* = 51.6, *p* < .001, *η* ^2^ *p* = 0.32	*F* = 0.9, *p* = .42*,* n.s.
Wrist peak velocity placement (ms)	**388 ± 10.9** ^a^	328 ± 11.4	345 ± 10.9	*F* = 7.2, *p* < .001, *η* ^2^ *p* = 0.06	*F* = 0.8, *p* = .48, n.s.
Index peak velocity (mm/s)	**1,128 ± 38.2** ^a^	1,366 ± 40.2	1,363 ± 38.5	*F* = 11.9, *p* < .001, *η* ^2^ *p* = 0.10	*F* = 0.5, *p* = .76, n.s.
Index peak velocity placement (ms)	**361 ± 11.3** ^a^	277 ± 11.5	290 ± 11.4	*F* = 18.8, *p* < .001, *η* ^2^ *p* = 0.12	*F* = 1.1, *p* = .35, n.s.
Time diff Index‐Wrist peak vel place (ms)	**−27 ± 5.6** ^a^	−51 ± 5.8	−55 ± 5.7	*F* = 6.9, *p* < .001, *η* ^2^ *p* = 0.06	*F* = 0.7, *p* = .59, n.s.
Wrist acceleration/deceleration phase (%)	46/54	45/55	**41/59** ^c^	*F* = 8.5, *p* < .001, *η* ^2^ *p* = 0.07	*F* = 1.9, *p* = .10, n.s.
Index acceleration/deceleration phase (%)	**43/57** ^a^	**38/62** ^b^	**35/65** ^c^	*F* = 22.5, *p* < .001, *η* ^2^ *p* = 0.17	*F* = 0.8, *p* = .48, n.s.
Wrist average velocity (mm/s)	**299 ± 9.3** ^a^	**411 ± 9.7** ^b^	360 ± 9.3	*F* = 33.6, *p* < .001, *η* ^2^ *p* = 0.23	*F* = 0.5, *p* = .71, n.s.
Index average velocity (mm/s)	**423 ± 15.8** ^a^	541 ± 16.4	525 ± 15.8	*F* = 15.7, *p* < .001, *η* ^2^ *p* = 0.14	*F* = 1.4, *p* = .23, n.s.
Wrist 3D distance (mm)	**260 ± 4.5** ^a^	305 ± 5.5	307 ± 4.5	*F* = 36.6, *p* < .001, *η* ^2^ *p* = 0.25	*F* = 3.4, *p* = .02, n.s.
Index 3D distance (mm)	**366 ± 5.2** ^a^	**403 ± 5.3** ^b^	**438 ± 5.3** ^c^	*F* = 46.5, *p* < .001, *η* ^2^ *p* = 0.29	*F* = 1.2, *p* = .29, n.s.
Grasp phase					
Grasp duration	77 ± 22.5	64 ± 24.3	**254 ± 22.6** ^c^	*F* = 18.9, *p* < .001, *η* ^2^ *p* = 0.15	*F* = 1.5*, p* = .19, n.s.
Transport‐to‐fit phase					
Transport‐to‐fit duration (ms)	1,461 ± 91	1,618 ± 92	**2,280 ± 93** ^c^	*F = *22.4, *p* < .001, *η* ^2^ *p* = 0.17	*F* = 9.8, *p* < .001, *η* ^2^ *p* = 0.15
Time transporting peg to goal (ms)	752 ± 31	691 ± 31	776 ± 32	*F* = 1.9, *p* = .14, n.s.	*F* = 2.7, *p* = .04, n.s.
Total peg rotation time (ms)	574 ± 48	659 ± 49	774 ± 49	*F = *4.2, *p* = .05, n.s.	*F* = 12.2, *p* < .001*,* *η* ^2^ *p* = 0.14
Wrist transport‐to‐fit MUs (*n*)	6.5 ± 0.7	7.5 ± 0.8	**13.3 ± 0.8** ^c^	*F = *22.1, *p* < .001, *η* ^2^ *p* = 0.16	*F* = 5.5, *p* < .001, *η* ^2^ *p* = 0.09
Index transport‐to‐fit MUs (*n*)	7.8 ± 0.7	9.7 ± 0.8	**15.4 ± 0.8** ^c^	*F* = 21.5, *p* < .001, *η* ^2^ *p* = 0.16	*F* = 9.8*, p* < .001, *η* ^2^ *p* = 0.15
Wrist average velocity (mm/s)	**181 ± 6.5** ^d^	212 ± 6.5	193 ± 6.5	*F* = 5.8, *p* < .005, *η* ^2^ *p* = 0.05	*F* = 11.3, *p* < .001, *η* ^2^ *p* = 0.17
Index average velocity (mm/s)	251 ± 9.0	273 ± 9.4	239 ± 9.1	*F* = 3.1, *p* = .05, n.s.	*F* = 9.4, *p* < .001, *η* ^2^ *p* = 0.14
Wrist 3D distance (mm)	**258 ± 7.2** ^a^	**315 ± 5.9** ^b^	**369 ± 7.2** ^c^	*F* = 58.1, *p* < .001, *η* ^2^ *p* = 0.34	*F* = 7.7, *p* < .001, *η* ^2^ *p* = 0.12
Index 3D distance (mm)	**359 ± 6.4** ^a^	**409 ± 5.4** ^b^	**437 ± 6.4** ^c^	*F* = 39.3, *p* < .001, *η* ^2^ *p* = 0.26	*F* = 12.8, *p* < .001, *η* ^2^ *p* = 0.18

Note

Significant (*p* < .005) age group differences (bolded) are indicated as (a) difference between adults and both child groups, (b) difference between 10‐year group and both adult and 6‐year group, (c) difference between 6‐year group and both adult and 10‐year group, and (d) difference between adults and 10‐year group.

Abbreviations: diff, difference; MUs, movement units; *n*, number; n.s., not significant; place, placement; vel, velocity.

#### 3.1.1 Latency phase

As shown in Table 1, a significant main effect of age was found for the latency. This effect was characterized by the adults showing a significantly longer latency time in comparison to the 6‐ and 10‐year groups, with the 10‐year‐olds displaying the shortest latency times. The longer latencies found in adults suggest action planning to optimize the initial grip formation in relation to the impending rotation and fitting of the peg into the goal‐slot.

#### 3.1.2 Reach‐to‐grasp phase

##### Duration

A significant main effect of age was found for duration of the reach‐to‐grasp phase (Table 1), mainly due to a longer reach‐to‐grasp duration for the 6‐year group compared with the adult‐ and 10‐year groups. The prolonged duration for the youngest children demonstrates online planning and adjustments in relation to the first goal (grasping of the peg), also in keeping with the relatively short onset latency for this group.

##### Wrist and index finger MUs

A significant main effect of age was also found for the number of wrist MUs during the reach‐to‐grasp phase (Table 1). The 6‐year group displayed significantly more MUs than both the adult and the 10‐year group, indicating less smooth (more segmented) wrist movement trajectories in line with increased online planning and adjustments. No similar significant main effect of age was found for the number of index finger MUs. However, the difference in number of MUs between index finger and wrist, independent of task, was larger for the adults (*M* = 1.8) and the 10‐year‐olds (*M* = 1.6) than for the 6‐year‐olds (*M* = 1.0), indicating that fingers/hand operated more isolated from the arm in the adults and 10‐year‐olds compared with being more coupled in the 6‐year‐olds.

##### Wrist and index finger peak velocity (mm/s)

For both the wrist and index finger peak velocity during the reach‐to‐grasp phase, a significant main effect of age was found (Table 1) in terms of the adults showing lower peak velocity than the 10‐ and 6‐year‐old children.

##### Time of wrist and index finger peak velocity (ms)

**Figure 3:**
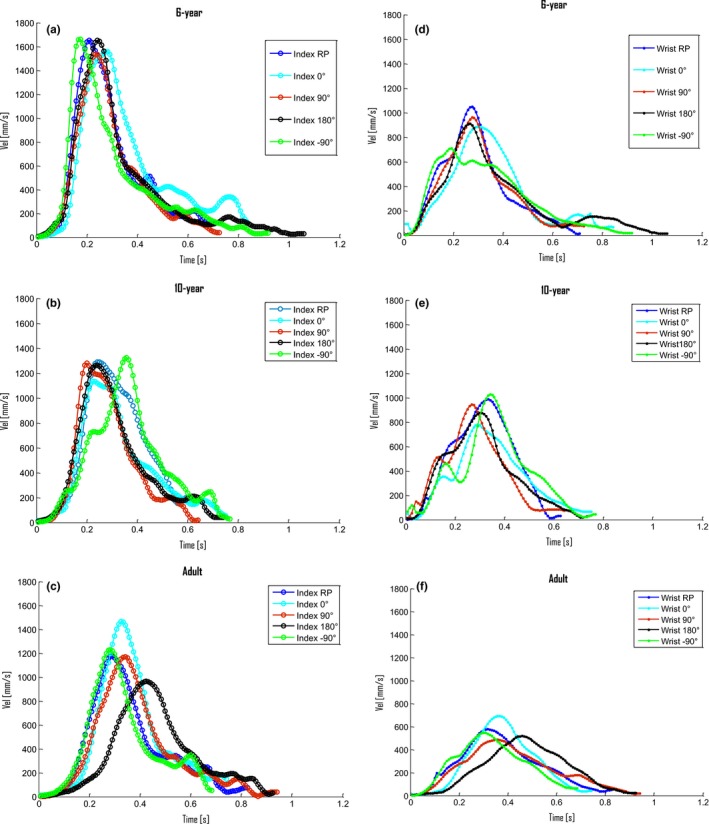
Examples of (a–c) index finger, and (d–f) wrist velocity profiles for the respective task condition, derived from one 6‐year‐old participant (a, d), one 10‐year‐old participant (b, e), and one adult (c, f)

In keeping with the peak velocity outcomes, the time of peak velocity for both the wrist and index finger was also significantly affected by age (Table 1). The timing of both wrist and index finger peak velocity was significantly later for the adults than for the 6‐ and 10‐year groups. Moreover, independent of task, there was a significant main effect of age in terms of less time difference between the index finger and wrist peak velocity for the adults (*M* = −27 ms, minus denoting that the index velocity peak is placed before the wrist peak) compared with both the 6‐ (*M* = −55 ms) and 10‐year old children (*M* = −51 ms). These outcomes indicate that the opening of the hand started earlier in the reach‐to‐grasp phase in the children compared with the adults. Figure 3 provides representative examples of reach‐to‐grasp index finger (a–c) and wrist (d–f) velocity profiles for the respective condition derived from each age group.

##### PPV (%)

A significant main effect of age was found for both the wrist and the index finger peak velocity placement (Table 1). In line with the prolonged total reach‐to‐grasp duration for the 6‐year group, PPV was earlier in the 6‐year group, followed by the 10‐year‐olds, and with the adults performing the relatively shortest deceleration phase. These findings suggest that the children, especially the 6‐year‐olds, required a relatively longer deceleration for the online planning and adjustments (stabilizing) of their grasp performance.

##### Wrist and index finger average velocity (mm/s)

In keeping with the finding of reduced peak velocity in the adults, a significant age effect for both the wrist and index finger average velocity was found (Table 1). Accordingly, on average, the adults moved both the wrist and the index finger significantly slower than both the 6‐ and the 10‐year‐old children. The 10‐year‐olds showed the highest wrist average velocity of all groups. Thus, also in keeping with the latency results, this is indicative of between‐group differences in speed‐accuracy trade off and of reduced planning for the onward goal in the children. In relation to the latter, it could be interpreted as the children, especially the 6‐year‐olds, using more implicit processing at the level of motor execution rather than explicit, second‐order task processing (movement planning).

##### Wrist and index finger 3D distance (mm)

Regarding the 3D distance for reach‐to‐grasp, a significant main effect of age was found for both the wrist and the index finger (Table 1). The adults displayed significantly shorter wrist 3D distances than both child groups. The 6‐year‐olds showed significantly longer index finger 3D distances than both adults and 10‐year‐olds. The difference between index finger and wrist was 106 mm (adult), 98 mm (10 years), and 131 mm (6 years). The prolonged distances add further support for inefficient planning strategies in the 6‐year‐olds, with the larger difference between finger and wrist reflecting either a wider hand opening or an indecisiveness in grip selection.

#### 3.1.3 Grasp phase

##### Duration

A significant main effect of age was found for the grasp phase (Table 1) in terms of considerably longer grasp durations for the 6‐year‐olds compared with the respective 10‐year‐ and adult group. Taken together with the above reported reach‐to‐grasp phase kinematics pertaining to the youngest children, the extended grasp phase suggests that the grasping preparation was less efficient in the 6‐year‐olds, possibly linked to reduced planning.

#### 3.1.4 Transport‐to‐fit phase

##### Duration

In agreement with the outcomes from the reach and the grasp durations, a significant main effect of age was found for the transport‐to‐fit duration (Table 1) in terms of a longer duration for the 6‐year‐olds in comparison to the adults and 10‐year‐olds, who did not significantly differ. This is indicative of less efficient movement control in the youngest children compared with the older children and adults. A significant main effect of task condition was also found (Table 1), characterized by longer duration for the 90° (*M* = 1,962 ms) and 180° (*M* = 2,341 ms) task conditions, compared with the RP (*M* = 1,378 ms), 0° (*M* = 1,517 ms), and −90° (*M* = 1,729 ms) tasks. However, no significant group by task interaction was found (*p* = .07).

##### Total peg rotation time

A significant main effect of task was revealed (Table 1), showing that the 90° (*M* = 817 ms), 180° (*M* = 798 ms) and −90° (*M* = 862 ms) all differed significantly from the RP condition (*M* = 418 ms), *p* = .0004; *p* = .0013; *p* = .00007, respectively, and from the 0° condition (*M* = 443 ms), *p* = .0015; *p* = .0038; *p* = .00028, respectively, by means of almost twice as long peg rotation times. There was no significant age by task interaction effect. When comparing total peg rotation time as the percentage of the total transport‐to‐fit duration, the adults spent a relatively greater part to rotate the peg (52% [298 ms]) in comparison to the 10‐ (45% [294 ms]) and the 6‐year‐olds (39% [303 ms]).

##### Wrist and index finger MUs

**Figure 4:**
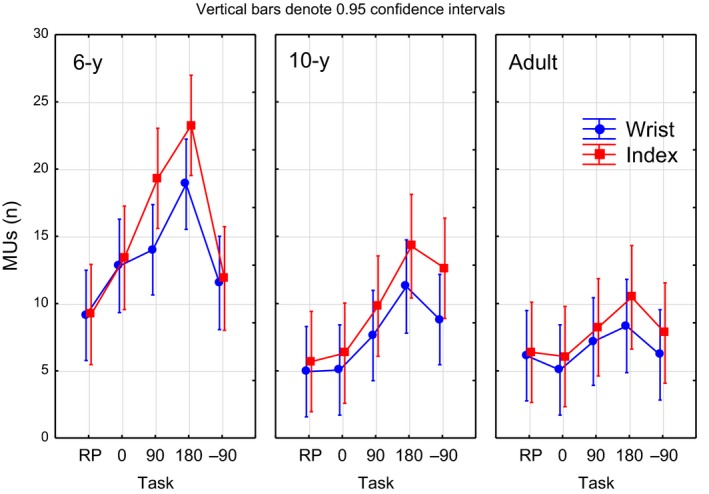
Mean wrist and index finger movement units (MUs) during transport‐to‐fit as a function of task condition for the different age groups

There was a significant main effect of age for both the number of wrist and index finger MUs (Table 1), characterized by the 6‐year‐olds displaying significantly more MUs than the adults and the 10‐year‐olds, who did not significantly differ. As for duration, these results are in line with the reach‐to‐grasp outcome and indicate less smooth movements and a greater need for movement adjustments in the 6‐year olds. A main effect of task was also found for both the number of wrist and index finger MUs (Table 1) in terms of significantly more MUs during the 180° condition than other task conditions (wrist: RP, *p* = .0008; 0°, *p* = .0006; index finger: RP, *p* = .000002; 0°, *p* = .002; −90°, *p* = .01; Figure 4). In addition, significantly more index finger MUs was found for 90° (*p* = .01) compared with RP. No significant age by task interaction effect was found for the number of index finger MUs (*F* = 1.9, *p* = .06). Notably, independent of age group, the majority of the transport‐to‐fit MUs originated from the final stage of the transport phase (i.e., fitting of the peg), corresponding to 88% (*M* = 3.9) for the adults; 86% (*M* = 4.5) for the 10‐year‐olds, and 76% (*M* = 8.0) of the total wrist MUs during transport‐to‐fit.

##### Wrist and index finger average velocity (mm/s)

For the wrist average velocity, a significant main effect of age was found (Table 1), exemplified by the adults displaying a significantly lower velocity than the 10‐year‐olds. Together with the longer distances, this further support a difference in speed‐accuracy trade off and motor planning between adults and 10‐year‐olds. The lack of velocity difference between the adults and the 6‐year group can be explained by less efficient movements and overall longer movement durations in the 6‐year‐olds generating lower average velocities, especially in the fitting part of the movement. The same effect of age for the index finger average velocity failed to reach significance (*p* = .026). A significant effect of task was, however, found for both the wrist and index finger velocity (Table 1). Independent of group, the average velocity was significantly lower for the respective 90° and 180° conditions compared with the other three task conditions.

##### Wrist and index finger 3D distance (mm)

**Figure 5:**
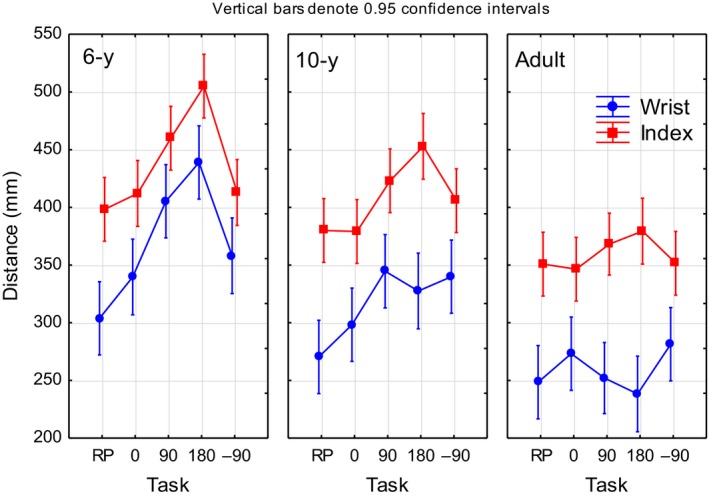
Mean wrist and index finger 3D distance during transport‐to‐fit as a function of task condition for the different age groups

A significant main effect of age was found for both the wrist and the index finger 3D distance (Table 1, Figure 5). All groups differed significantly from each other in terms of the adults performing the shortest 3D distances, followed by the 10‐year group, and lastly the 6‐year group showing the longest 3D distances. Also, a significant main effect of task was found for the 3D distance of both the wrist and index finger (Table 1). The wrist 3D distances for 90°, 180° and −90° were significantly longer than those for the RP and 0° conditions, and the index finger 3D distance for 180° was significantly longer than for all other task conditions. A significant age by task interaction was found for the wrist (*F* = 4.09, *p* = .0049). For the 180° task, the 6‐year‐olds showed longer 3D distances than both the adults and the 10‐year‐olds for all task conditions. The 6‐year‐olds also showed significantly longer wrist 3D distances for 90° compared to the adults, and to the 10‐year‐olds for RP, independent of task. Moreover, a main effect of group was found regarding the average 3D distance between the wrist and the index finger (*F* = 8.68, *p* = .0002, *η* ^2^ *p* = 0.07). The distance was longest for the adults (*M* = 101 mm), followed by the 10‐year‐olds (*M* = 98 mm), and shortest for the 6‐year‐olds (*M* = 68 mm). Post hoc testing showed that this difference was significant between the 6‐year‐olds and adults (*p* = .0004), and between 6‐ and 10‐year‐olds (*p* = .007), whereas the adults and 10‐year‐olds did not significantly differ (*p* = .55). Notably, differences were particularly apparent for the 180° condition (*M*_Adults_ = 144 mm; *M*_10‐year_ = 113 mm; *M*_6‐year_ = 66 mm). Thus, these outcomes indicate a greater independence between the index finger and wrist for the adults and 10‐year‐olds during transport‐to‐fit, likely related to autonomous index finger adjustments during peg rotation in hand, as opposed to more simultaneous rotating of wrist and index finger in the 6‐year group.

### 3.2 Correlation analyses

Table 2 shows outcomes of correlation analyses between reach‐to‐grasp/grasp and transport‐to‐fit parameters for the respective age group. Table 3 shows the correlations related to the rotations made within the transport‐to‐fit phase for the respective age group.

**Table 2:** Correlations between kinematic variables derived from the RTG phase and the TTF phase

	TTF duration	TTF wrist MU	TTF wrist mean velocity	TTF wrist distance
6‐year				
Latency	0.174	0.160	−0.315	−0.182
RTG duration	**0.526**	**0.570**	**−0.572**	0.024
RTG wrist MU	**0.456**	**0.532**	**−0.462**	0.013
RTG wrist peak velocity	−0.220	−0.204	**0.436**	0.172
RTG wrist peak placement	**0.375**	**0.371**	**−0.479**	0.044
RTG deceleration	**0.331**	0.322	**−0.374**	0.003
RTG wrist mean velocity	**−0.483**	**−0.440**	**0.687**	0.061
RTG wrist distance	0.263	0.320	−0.171	0.240
Grasp duration	**0.602**	**0.568**	**−0.555**	0.106
10‐year				
Latency	0.047	−0.053	−0.172	−0.201
RTG duration	0.238	0.064	**−0.458**	−0.109
RTG wrist MU	0.048	−0.018	−0.135	0.002
RTG wrist peak velocity	−0.312	−0.187	**0.531**	0.103
RTG wrist peak placement	0.175	−0.024	−0.324	−0.110
RTG deceleration	0.079	0.150	−0.127	0.061
RTG wrist mean velocity	−0.204	−0.033	**0.459**	0.118
RTG wrist distance	0.118	0.072	−0.133	−0.014
Grasp duration	0.026	−0.120	−0.075	0.013
Adult				
Latency	0.171	−0.027	−0.157	0.043
RTG duration	**0.407**	0.244	**−0.371**	0.068
RTG wrist MU	0.207	0.276	−0.238	0.012
RTG wrist peak velocity	−0.075	0.019	0.308	0.279
RTG wrist peak placement	0.323	0.227	−0.309	0.012
RTG deceleration	0.165	0.079	−0.111	0.094
RTG wrist mean velocity	−0.279	−0.106	**0.484**	0.254
RTG wrist distance	0.294	0.226	0.003	**0.397**
Grasp duration	0.054	0.004	−0.140	−0.077

Note

Bolded values are significant at *p* < .005.

Abbreviations: MU, movement units; RTG, reach‐to‐grasp; TTF, transport‐to‐fit.

**Table 3:** Correlations between rotation phases I (peg transporting) and II (peg fitting) and parameters derived from the transport‐to‐fit phase

	TTF duration	TTF wrist MU	TTF wrist mean velocity	TTF wrist distance	TTF peg MU	Goal residual angle
6‐year						
Rota I	**0.544**	**0.521**	**−0.529**	0.123	**0.519**	**0.469**
Rota II	**0.915**	**0.812**	**−0.594**	**0.696**	**0.911**	**0.361**
10‐year						
Rota I	**0.528**	**0.451**	**−0.377**	**0.656**	**0.598**	0.158
Rota II	**0.650**	**0.590**	**−0.506**	**0.567**	**0.647**	0.181
Adult						
Rota I	**0.558**	0.325	−0.236	**0.397**	**0.541**	**0.357**
Rota II	**0.666**	**0.466**	**−0.423**	0.263	**0.578**	0.304

Note

Bolded values are significant at *p* < .005.

Abbreviations: MU, movement units; Rota, rotation phase in seconds; TTF, transport‐to‐fit.

#### 3.2.1 Associations between reach‐to‐grasp/grasp and transport‐to‐fit

In all age groups, there were associations between mean wrist velocity of the reach‐to‐grasp and transport‐to‐fit phases. Furthermore, there were associations between number of MUs during the reach‐to‐grasp and transport‐to‐fit phases. This demonstrates that the two action phases are coupled and planned at each age. For adults, longer reach‐to‐grasp duration was significantly related to both longer transport‐to‐fit duration and lower mean wrist velocity during transport‐to‐fit. Longer reach‐to‐grasp duration was also associated with lower mean wrist velocity during transport‐to‐fit for the 10‐year‐olds, in addition to a positive correlation between wrist peak velocity during reach‐to‐grasp and wrist mean velocity during transport‐to‐fit. A wide range of correlations between reach‐to‐grasp and transport‐to‐fit kinematics was revealed for the 6‐year‐olds (Table 2), inferring a stronger coupling between movement performance in these phases for the younger children compared with older children and adults.

### 3.3 Rotation analyses

#### 3.3.1 Rota I and II

**Figure 6:**
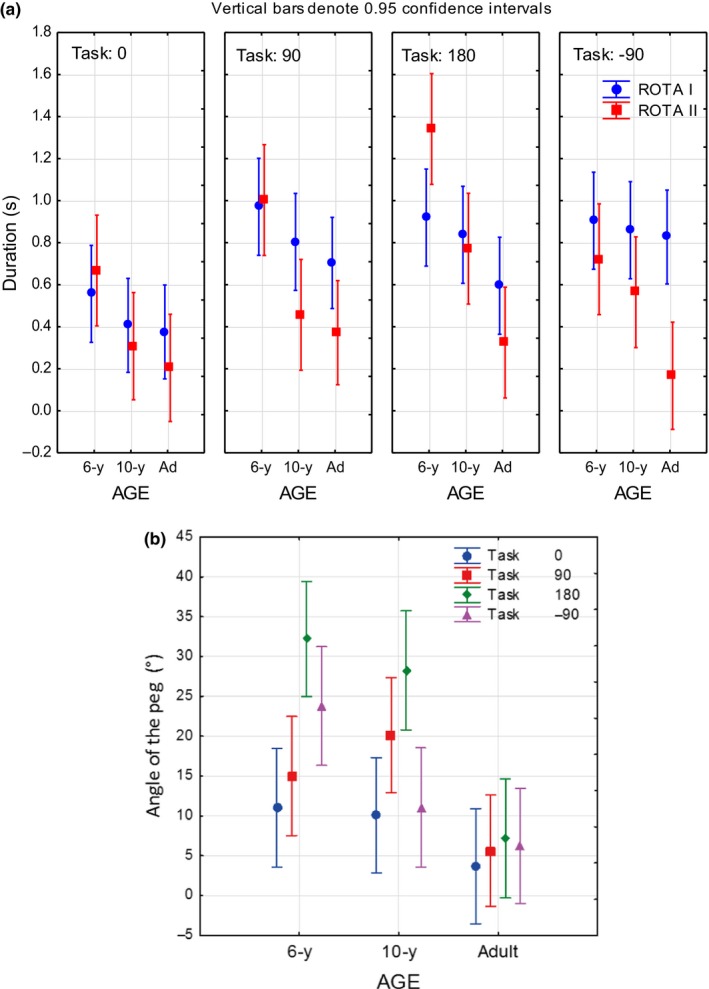
Figure depicting (a) the relation between the rotation parameters Rota I (peg transport) and Rota II (peg fitting) mean durations with respect to age groups and task conditions, and (b) the mean angle of the horizontal line of the peg relative the frontoparallel axis at goal arrival for the different task conditions as a function of age

To analyze the effect of age and task on the duration of the two identified rotation phases (Rota I and II), a mixed analysis of variance (MANOVA) with repeated measures was used, with age and task as the between‐group factors and Rota I (peg transport phase) and II (peg fitting phase) as repeated factor. A significant main effect of age was found for the rotation durations (*F* = 17.55, *p* < .001, *η* ^2^ *p* = 0.16). This effect was characterized by the 6‐year‐old children showing significantly longer rotation times compared with both the 10‐year group and the adults. This was particularly evident for Rota II, where the 6‐year‐olds showed longer rotation durations (*M* = 940 ms) than the 10‐year‐olds (*M* = 530 ms) and the adults (*M* = 270 ms). A significant main effect of task was also found (*F* = 7.29, *p* < .001, *η* ^2^ *p* = 0.11), and a significant task by repeated Rota I and II interaction (*F* = 5.78, *p* < .001, *η* ^2^ *p* = 0.09). As illustrated in Figure 6a, these effects were mainly related to longer rotation times in the 6‐year‐olds during the 90° and 180° conditions. The 6‐year‐olds also had longer rotation times for Rota II than the 10‐year‐olds and adults in the task conditions 0°, 90° and 180°.

#### 3.3.2 Angle of the peg at goal

With regard to the angle of the peg in relation to the goal angle at the end of the transport phase (Rota I), a significant main effect of age was found (*F* = 17.91, *p* < .001, *η* ^2^ *p* = 0.17), characterized by the adults showing overall smaller angle differences between the peg and the goal (*M* = 5.6°) than both the 6‐year‐olds (*M* = 20.7°) and the 10‐year‐olds (*M* = 17.3°). This finding indicates a more consistent pro‐active peg rotation and more reliable motor planning ability in adults than children. A significant main effect of task was also found (*F* = 7.6, *p* < .001, *η* ^2^ *p* = 0.11) by means of overall significantly larger angle differences in the 180° task condition (*M* = 22.7°) compared with the other task conditions (Task 0°, *M* = 8.2°; Task 90°, *M* = 13.4°; Task −90°, *M* = 15.5°). Figure 6b shows that this main effect of task condition is solely linked to the two child groups (and particularly prominent in the task condition 180°), however, no significant age by task interaction effect was found (*F* = 2.1, *p* = .06, *η* ^2^ *p* = 0.05).

#### 3.3.3 Correlations within the transport‐to‐fit phase

As shown in Table 3, Rota I and II durations were associated with transport‐to‐fit kinematics for all age groups. First, rotation durations were strongly associated with transport‐to‐fit duration and peg MUs. Second, transport‐to‐fit mean velocity was negatively associated with rotation durations except for Rota I in adults. There were strong relations between Rota II and transport‐to‐fit durations, wrist MUs, and distances for the 6‐ and 10‐year‐olds, supporting the fact that the children, the youngest in particular, were prone to save their rotating efforts to the very end task and had to perform multiple corrective actions to finalize it. The absence of significant correlations for adults in Rota I indicates that the action was more dominated by the index finger than the wrist.

### 3.4 Video coding

#### 3.4.1 End state comfort

For the 6‐year group, 29.8% of the trials (7 in the 90°, 5 in the 180°, and 2 in the −90° rotation) were judged as no ESC. Three children consistently showed ESC, four inconsistent ESC (at least one trial no ESC), and one child did not show ESC in any of the trials. In the 10‐year group, 12.5% of the trials were considered as no ESC (all in the 90° rotation). Five 10‐year‐olds showed inconsistent ESC. All adults showed complete ESC. To be noted is that, on a group level, the kinematic outcomes for trials judged as no ESC, mainly found in the 6‐year group, did not differ in any meaningful way from trials with ESC. Thus, the main results of this study is not primarily related to distortion by the children displaying incomplete ESC.

#### 3.4.2 Goal interpretation errors

In the 6‐year group, goal interpretation errors were noted in 7.7% of the trials (3 in the 0°, 2 in the 90°, and 1 in the −90° rotation). Three children showed at least one error. For the 10‐year group, goal interpretation errors were shown in 10% of the trials (3 in the 0°, 1 in the 90°, 1 in the 180°, and 3 in the −90° rotation). Three children displayed at least one error. Within a total of 80 trials, one goal interpretation error was committed in the adult group (in the 90° rotation).

#### 3.4.3 Type of grip

11.5% of the trials in the 6‐year group were characterized by an inefficient grip strategy. Five children consistently used efficient grip types over the trials, three displayed an inconsistent grip strategy. For the 10‐year‐old children, 12.5% of the trials were categorized as inefficient grip. A consistent efficient grip strategy was shown by five children and three showed inconsistency. All adults consistently employed an efficient grip strategy.

## 4 DISCUSSION

Age‐related differences in manual motor planning were investigated in typically developing children at 6 years, 10 years, and adults. The participants were asked to reach for and move a semicircular peg between two positions to fit it into a semicircular goal slot. The task was dependent on second‐order planning, that is, the peg had to be rotated different amounts to fit into the slot and this rotation had to be prepared differently depending on the condition.

As expected (e.g., Kuhtz‐Buschbeck et al., 1998), significant differences in kinematic outcomes between the groups were found at each of the four movement phases investigated (onset latency, reach‐to‐grasp, grasp, transport‐to‐fit). In accordance with the suggested developmental trend for action organization (Jongbloed‐Pereboom et al., 2013; Rosenbaum et al., 2012; Scharoun Benson et al., 2018; Stöckel et al., 2012; van Swieten et al., 2010; Wilmut & Byrne, 2014; Wunsch et al., 2016), less efficient movement organization related to task performance was found in the children than in the adults. More specifically, the 10‐year‐olds mainly differed from the adults regarding kinematic parameters associated with motor planning, whilst the 6‐year‐olds differed regarding parameters related to both motor planning and control.

### 4.1 Reach‐to‐grasp phase

According to the planning‐control model for first‐order goal‐directed reaching movements (Glover, 2004, Glover, Wall, & Smith, 2012), the onset latency phase is where the action planning occurs, that is, processes related to determining the action goal, identifying and selecting the target, analyzing object affordances, and timing. After initiation of the movement, online control processes involving integration of sensory feedback and feed‐forward mechanisms monitor the movement execution. Overall, we found that adults displayed longer onset latencies than children, suggesting more action planning in the adults. In support of this, reduced average velocities and later time to peak velocities (including indications of later grip opening) were also observed in the adults. In reach‐to‐grasp movements not requiring onward action, adults typically display a reversed pattern compared with children (e.g., Kuhtz‐Buschbeck et al., 1998; Olivier et al., 2007). Thus, a reasonable interpretation is that the adults’ reach‐to‐grasp performance in this study was affected by attention to the second‐order goal. For example, assuming that the nature of the present task may introduce a certain ambiguity regarding the motor goal even after the movement has started, the finding of reduced reaching velocity could reflect an intermediate movement strategy in the adults. This is related to a dedicated motor plan for uncertain goals that is typically generated at slower speeds to promote better task performance (Wong & Haith, 2017).

Still, similar to previous kinematic studies, the adults performed better organized reach‐to‐grasp movements than the children in terms of shorter durations, smoother (less segmented) wrist movement trajectories, better proportioned accelerations/decelerations, and shorter 3D distances. Notably, differences were more evident between adults and 6‐year‐olds than between adults and 10‐year‐olds. Thus, online control and adjustments related to grasp performance were more challenging for the children, the younger ones in particular. Olivier et al. (2007) reported that reaching and grasping is not yet well linked at 6 years, and at 11 years a better developed coupling between reaching and grasping can be observed (although not yet comparable to adults). Furthermore, the transition between 6‐ and 10‐years was regarded as critical in terms of the appearance of feedback‐based reaching‐grasping coordination. Interestingly, children at 8 years have also been reported to more consistently employ anticipatory coordination both during goal‐directed reaching (Kuhtz‐Buschbeck et al., 1998; Wilson & Hyde, 2013) and in lifting tasks (Forssberg, Eliasson, Kinoshita, Johansson, & Westling, 1991). The reach‐to‐grasp performance of the children in this study agrees well with the outcomes from studies using single manual actions. Taken together, this suggests that particularly the youngest children did not fully plan ahead for the second‐order goal but focused initially on grasping the peg to subsequently consider the second goal of fitting the peg. This notion of partition is supported by the short onset latency times (indicating less preplanning), and increased average velocities, earlier time to peak velocities, and a proportionally shorter reach‐to‐grasp phase compared with adults (conceivably linked to reduced preplanning).

Differences in kinematic outcome depending on goal requirements during reach‐to‐grasp would provide information about whether reaching actions were specific to the rotation condition. However, such differences were not evident for any of the age groups. Previous studies involving onward actions in children and adults are not consistent in reporting kinematic differences in the initial reaching sequence (Johnson‐Frey, McCarty, & Keen, 2004; Kuhtz‐Buschbeck et al., 1998; Wilmut et al., 2013; Zoia et al., 2006). This is likely due to task differences (Wilmut et al., 2013). The relatively challenging task used in this study may thus have imposed certain constraints that were not revealed by the reach‐to‐grasp kinematics in a clear‐cut way. For example, many studies reporting differences in initial reaching kinematics have contrasted precise and imprecise secondary tasks, while all conditions in this study required precision.

### 4.2 Grasp phase

One striking result was the much longer grasp durations for the 6‐year‐olds than for the other groups. In fact, the grasping time for the 6‐year‐olds were three times longer than for the 10‐year‐olds and the adults. The adults generally displayed a decreased time difference between the index finger and wrist time to peak velocity during reaching, indicating a late opening of the hand while still ending up with a swiftly formed, efficient grip. Both adults and 10‐year‐olds showed less agreement between wrist and index finger MUs during reaching than the 6‐year group. This suggests a more mature and independent grasping preparation. The findings are similar to previous studies in terms of the younger children displaying less mature grasping and the older children showing better organized, but not yet fully mature, grip formation and adaptation (e.g., Kuhtz‐Buschbeck et al., 1998; Olivier et al., 2007).

The extended grip formation period found for the 6‐year group is interpreted as less efficient preplanning, online monitoring and adjustments during deceleration. In contrast, they were earlier in opening the hand and relied more on visual information. This indicates that the 6‐year‐olds focused on the initial task and had less forward control of the rotation task. In addition, the longer reaching and grasp durations in the 6‐year‐olds support the suggestion that these children were prone to use a step‐wise strategy. This interpretation is in line with previous reports of longer reach‐to‐grasp durations for first‐order tasks without onward action than for second‐order tasks requiring a subsequent action to achieve the action goal (Gentilucci, Negrotti, & Cangitano, 1997; Johnson‐Frey et al., 2004).

### 4.3 Transport‐to‐fit phase

In keeping with findings related to the reach‐to‐grasp phase, the youngest children performed less efficiently organized transport‐to‐fit movements than both the older children and the adults. They had longer durations, more segmented movement trajectories, and longer 3D distances. The adults successfully performed the tasks using smoother and straighter transport‐to‐fit movements at lower average velocities than the children, suggesting a better speed‐accuracy trade‐off (likely related to appropriate planning).

Importantly, there are two strategies during transport‐to‐fit. The adults rotated the peg while transporting it toward the goal. In contrast, the children made supplementary and corrective rotations after the hand had arrived at the goal (Rota II). This was especially characteristic for the 180° rotation (see Figure 6a). 3D distances for both wrist and index finger were found to be longer in the more challenging rotational conditions, the 180° condition in particular. Furthermore, the average 3D distances between the wrist and index finger trajectories were longest for the adults and shortest for the 6‐year group, particularly for the 180° condition. Thus, the adults and the 10‐year‐olds moved the index finger more independent of the wrist (rotating peg in hand) to promote an efficient transport‐to‐fit phase, and used such a strategy more during more demanding task conditions. The 6‐year group additionally displayed more segmented and corrected movements than the 10‐year and adult groups in the more complex goal conditions during this phase.

Thus, the consistent pro‐active peg rotation among the adults indicates a reliable motor planning ability, supporting controlled arrival at the goal and a comfortable fitting of the peg. The children did not rotate the peg during transport to the same extent, and subsequently spent longer time fitting the peg into the goal slot. Therefore, in keeping with the video outcome concerning problems with ESC, both groups of children showed similar planning difficulties during transport, although more distinctly in the 6‐year‐olds. The larger amount of corrected movements during transport‐to‐fit (largely corresponding to the duration of fitting) in the 6‐year group further suggests that the planning difficulties displayed by the younger children appear to be amplified by less independent finger‐wrist movements and reduced online control.

Overall, there are three important findings regarding the segmentation in terms of MUs. First, the number of MUs is related to how difficult and complex the task is and how much forward planning that is accomplished. The results confirm that between 6 and 10 years of age there is a distinct improvement of skill level (Contreras‐Vidal, 2006). Second, it also shows that the number of MUs is much larger for the transport‐to‐fit phase than for the reach‐to‐grasp, especially at the final corrections (Rota II). Finally, the increase in rotational demands is related to increase in number of wrist MUs (Table 3).

Another consideration related to the performance of the different age groups is how the different parts of the movement correlate with each other. An adaptation of the planning‐control model to second‐order planning predicts that the initial reach‐to‐grasp movement may involve planning for the next phase of the action. The efficiency of this part will be influenced by prior planning during the initial reach‐to‐grasp phase. Thus, correlations between relevant outcomes of these action phases would indicate that efficiency of action during reach‐to‐grasp is related to efficiency of onward action performance. Here, the most striking finding was the multitude of correlations between reach‐to‐grasp and transport‐to‐fit kinematics in the 6‐year group compared with older children and adults. Thus, the 6‐year‐olds showed a robust coupling between the two movement phases. This suggests that these two phases are jointly programmed in the 6‐year‐olds but not in the older age groups.

### 4.4 Conclusions

This study shows that a quantified analysis of action development related to second‐order motor planning in children reveals many important changes over age. While all participants mastered the task of grasping, transporting, and rotating the pegs, they did it at different skill levels. The adults rotated the peg during transport while the children did so mostly after reaching the goal. It is concluded that quantitative analysis of reach‐to‐grasp‐to‐fit movements with various task constraints gives insights into typical development of manual actions. Here, this included shorter onset latency times, higher velocities, earlier time to peak velocities during reach‐to‐grasp in the children, longer grasp durations in the 6‐year‐olds, and age‐related task effects on movement segmentation and peg rotation during transport‐to‐fit. This study also opens a window onto the problems experienced by children at risk for motor planning deficiencies like those expressed by children with autism spectrum disorder (von Hofsten & Rosander, 2012). Increased understanding of the relations between action execution, sequential motor planning and underlying movement kinematics may also aid in tailoring relevant interventions for children with manifested motor planning problems.

## CONFLICT OF INTEREST

The authors have no conflict of interest to declare.

## Data Availability

Data are available upon reasonable request.
